# Low serum magnesium levels and diabetic foot ulcers

**DOI:** 10.12669/pjms.296.3978

**Published:** 2013

**Authors:** Şakir Özgür Keşkek, Sinan Kırım, Adil Karaca, Tayyibe Saler

**Affiliations:** 1 Şakir Özgür Keşkek, MD, Department of Internal Medicine, Numune Education and Research Hospital, Adana, Turkey.; 2 Sinan Kırım, MD, Department of Internal Medicine, Numune Education and Research Hospital, Adana, Turkey.; 3 Adil Karaca, MD, Department of Internal Medicine, Numune Education and Research Hospital, Adana, Turkey.; 4 Tayyibe Saler, MD, Department of Internal Medicine, Numune Education and Research Hospital, Adana, Turkey.

**Keywords:** Diabetic foot ulcer, Magnesium, Patients with diabetes

## Abstract

***Objective:*** Magnesium plays an important role in glucose homeostasis and insulin sensitivity. The aim of this study was to investigate the association between serum magnesium levels and diabetic foot ulcers.

***Methods:*** A total of 147 subjects were included in this study. The participants were divided into three groups, including a study group of 49 patients with diabetes and foot ulcers, a control group of patients with diabetes without foot ulcers and a control group of 49 healthy subjects. Measurements and comparisons were made of the participants’ magnesium levels, HBA1C percentages, serum fasting glucose levels, creatinine levels and serum lipid levels for all groups. MedCalc version 12.0 (MedCalc, Turkey) was used for the statistical analysis.

***Results:*** The groups were similar in terms of age and sex (p=0.116 and 0.897, respectively). The magnesium levels of the patients with diabetes and foot ulcers were lower than those in the patients with diabetes without foot ulcers and the healthy subjects (p<0.001). There was a strong relationship between the serum magnesium levels and the incidence of diabetic foot ulcers (OR 5.9, Cl 95% 2.7-12.6, p<0.001).

***Conclusions:*** A low serum magnesium level is associated with diabetic foot ulcers. Therefore, the magnesium levels should be controlled in patients with diabetes (with or without foot ulcers) and magnesium supplementation can be a complimentary treatment in such cases.

## INTRODUCTION

Diabetes mellitus (DM) is a common metabolic disease that affects individuals worldwide. DM has become an important public health concern because of its increasing prevalence, chronic nature and serious complications.^1^ Diabetic foot ulcers are one important complication of diabetes, and are a leading cause of hospital admissions for people with DM in developed countries, as well as being a major morbidity associated with diabetes.^2^ The pathophysiology of chronic diabetic foot ulcers is complex and incompletely understood; however, micro- and macroangiopathy, as well as neuropathy, strongly contribute to the development and delayed healing of diabetic wounds.^3^

Magnesium is one of the major cations found in plasma. It regulates ion channels and has co-enzymatic activity in more than 300 enzymatic reactions that affect the energetic metabolism and synthesis of protein. It also plays an important role in neuromuscular transmission. Extracellular magnesium levels contribute only a small part to the body’s total magnesium, but they do have a good correlation with intracellular magnesium.^4^^-^^7^

Magnesium deficiencies in type 2 DM have been shown in several studies,^8^^-^^11^ and a low magnesium level can impair glucose homeostasis and insulin sensitivity in these patients.^8^^,^^12^ On the other hand, the Hisayama study has shown that magnesium intake decreases type 2 diabetes risk through the improvement of insulin resistance and inflammation.^13^ Complications of DM develop when tissues are chronically exposed to high blood glucose levels. The non-enzymatic glycosylation of proteins, and the accumulation of the sorbitol can cause irreversible changes in the tissues. Additionally, both microvascular and macrovascular complications can develop. Neuropathy, which is a risk factor for the progression of diabetic foot ulcers, can also develop by this mechanism.^14^

The objective of this study was to investigate serum magnesium levels in patients with DM and foot ulcers, and assess the relationship between serum magnesium levels and diabetic foot ulcers.

## METHODS

This cross-sectional study was carried out in the internal medicine outpatient clinics of the Adana Numune Training and Research Hospital in Turkey, from 11 July, 2011, to 20 March, 2012. The institutional review board of the hospital approved this experiment, and informed consent was obtained from all subjects. All procedures were followed in accordance with the ethical standards of the Responsible Committee on Human Experimentation, and with the Helsinki Declaration of 1975, as revised in 2008.

A total of 147 subjects were included in this study and divided into three groups. The study group consisted of 49 patients with diabetes and foot ulcers, and the control groups consisted of 49 patients with diabetes without foot ulcers and 49 healthy subjects, respectively.

Subjects on medication that affected magnesium levels, with a history of diabetic foot ulcers for more than three months, hypertension, renal failure, heart failure, acute or chronic diarrhoea, malabsorption, thyroid or adrenal dysfunction, malignancies, sepsis, a history of alcohol intake or smoking, and who were pregnant or lactating were not included in this study.

The diagnoses of diabetic foot ulcers were established by the presence of foot ulcerations with full-thickness skin defects, whose recovery time was longer than 14 days.

Measurements were taken of the participants’ serum magnesium levels, blood glucose levels, HBA1C percentages, serum lipid levels, ASTs, ALTs, creatinine levels and complete blood counts. Venous blood samples were collected after overnight fasting. Magnesium levels were analysed using the colorimetric method, with the Roche C-501 (Japan), and the reference range was between 1.9-2.6 mg/dl. Serum glucose, lipids, ASTs, ALTs and creatinine levels were analysed on the Beckman Coulter Synchron LX 20 (Massachusetts, USA), using commercially available kits. Complete blood counts were measured by using fluorescence flow cytometry on the Sysmex XE 2100i (Japan). HBA1C percentages were analysed using high-performance liquid chromatography.

The blood pressures of the subjects were measured after 10 minutes of rest with periodically calibrated sphygmomanometers (Erka, Germany) at least two times on two different days. Hypertension was diagnosed by a systolic blood pressure ≥ 140 mmHg, or a diastolic blood pressure ≥ 90 mmHg. Patients with higher blood pressure were excluded from the study.

The MedCalc 12.0 software program (MedCalc, Turkey) was used for the statistical analysis, and data were reported as the mean ± sd. Chi square and Kolmogorov-Smirnov tests were used to compare the categorical measurements between the groups, and to show the normal distribution of the quantitative measurements, respectively. The independent groups t-test was used for the comparison of the quantitative measurements between the two groups, and the ANOVA or Kruskal-Wallis tests were used to compare the quantitative measurements between the three groups. A correlation coefficient was used to analyse the degree of association between the two variables (Pearson correlation coefficient (r), with P-value and 95% CI for r.). A log transformation was used for the variables that were not normally distributed, and an odds ratio was used to analyse the degree of association between the serum magnesium levels and diabetic foot ulcers. The level of statistical significance was 0.05.

## RESULTS

Groups were similar in terms of age and sex (p=0.116 and 0.897, respectively), and there were 24 women (48.9%) and 25 men (51%) in the study group. The control group of patients with diabetes consisted of 26 women (53.1%) and 23 men (46.9%), while the control group of healthy participants contained 24 women (48.9%) and 25 men (51%). There was no statistically significant differences between the duration of DM in the patients with diabetes and with or without foot ulcers (p=0.786). The mean duration of the diabetic foot ulcers was 27.4±9.2 days (Table-I).

**Table-I:** Demographic characteristics of the groups

	*Diabetes Mellitus with foot ulcer* *N=49*	*Diabetes Mellitus without foot ulcer* *N=49*	*Control group* *N=49*	*p*
Age (years)	56.6±8.6	53.4±11.4	53.1±11.4	0.116
Female N (%)	24 (48.9%)	24 (48.9%)	26 (53.1%)	0.897
Diabetes duration (years)	12.4±5.0	12.1±4.3	----	0.786
Diabetic foot ulcer duration (days)	27.4±9.2	----	----	----

**Table-II:** Magnesium levels and other biochemical measurements of the groups

	*Diabetes Mellitus with foot ulcer* *N=49*	*Diabetes Mellitus without foot ulcer* *N=49*	*Healthy group* *N=49*	*p*
Serum fasting glucose (mg/dl)	221.5±85.2	184.9±65.6	90.1±10.4	<0.001
HBA1C %	10.0±1.8	8.5±1.6	5.5±0.2	<0.001
Triglyceride (mg/dl)	216.2±109.9	178.1±90.4	139.7±54.9	0.007
HDL (mg/dl)	39.2±8.7	41.4±8.9	44.3±8.6	0.019
LDL (mg/dl)	110.9±30.9	119.3±39.1	125.2±34.2	0.129
Creatinine (mg/dl)	0.84±0.23	0.86±0.21	0.82±0.16	0.669
Magnesium (mg/dl)	1.73±0.19	1.91±0.12	2.35±0.27	<0.001

**Table-III:** The correlation between magnesium and serum fasting glucose, HBA1C, HDL, duration of diabetes and duration of diabetic foot ulcer

		*Magnesium*
Serum fasting glucose	r p	-0.518 <0.001
HBA1C	r p	-0.579 <0.001
HDL	r p	0.254 0.002
Diabetes duration	r p	-0.000 0.996
Diabetic foot ulcer duration	r p	0.019 0.895

The magnesium levels of the patients with diabetic foot ulcers were lower than in those patients with diabetes without foot ulcers and in the healthy subjects (p<0.001). The serum fasting glucose levels, HBA1C percentages, triglycerides and HDL levels of the groups were statistically different (p<0.001, <0.001, =0.007 and =0.019, respectively). There were no statistically significant differences between the serum LDL and creatinine levels (p=0.129 and 0.669, respectively) (Table-II). There was an inverse correlation between the magnesium levels and serum fasting glucose levels and HBA1C percentages (p<0.001 for each), and the HDL levels were correlated with the magnesium levels (p=0.002, r=0.254). There was no correlation between the magnesium levels and the duration of DM or duration of diabetic foot ulcers (P>0.05) (Table-III); however, there was a strong association between the serum magnesium levels and diabetic foot ulcers (OR 5.9, Cl 95% 2.7-12.6, p<0.001).

## DISCUSSION

In this study we detected a strong association between low magnesium levels and diabetic foot ulcers. Additionally, magnesium levels of the subjects are inversely correlated with serum fasting glucose levels and HBA1C percentages. The results of this study are in agreement with the literature and the results of several recent studies.^8^^,^^15^^-^^17^

Magnesium is an essential element that plays an important role in many biological functions, and low magnesium levels are associated with insulin resistance, type 2 DM, dyslipidaemia and hypertension.^7^^,^^18^^,^^19^ Many studies have shown that the plasma levels of magnesium are lower in patients with type 2 DM.^8^^,^^11^^,^^20^^,^^21^ One of the causes of low magnesium in type 2 diabetes is increased renal excretion due to hyperglycaemia, glycosuria and insulin resistance; additionally, low magnesium can be caused by reduced intestinal absorption due to diabetic autonomic neuropathy.^15^^,^^22^

Low intracellular magnesium levels negatively affect the transportation of cellular glucose, tyrosine kinase activity, post-receptor insulin action and secretion of insulin from the pancreas.^22^^,^^23^ In a study by Rodriguez-Moran and Guerrero-Romero, post-receptor insulin resistance was related to the TNF-alpha concentration, which is associated with low serum magnesium levels.^24^

Considering the pathophysiological mechanisms, it can be said that low serum magnesium concentration is a risk factor for developing type 2 DM, and that type 2 DM is one of the causes of hypomagnesaemia. Hypomagnesaemia can worsen the glycaemic control in DM, and both micro- and macrovascular complications of diabetes are strongly associated with hyperglycaemia and/or uncontrolled glycaemia.

The HDL levels were low and triglyceride levels were high in the study group when compared to the control groups. As a cardiovascular risk factor, HDL is correlated with magnesium levels, and in another Guerrero-Romero and Rodriguez-Moran study, they found an association between low magnesium and HDL levels.^25^ We believed that the high HBA1C percentages and triglyceride levels in the present study were dependent on insulin resistance, hyperglycaemia, or uncontrolled diabetes mellitus due to hypomagnesaemia. Further reinforcing this link, high HBA1C percentages and triacylglycerol levels in patients with diabetes mellitus, and low magnesium levels were reported by the Srinivasan et al. study. Additionally, Rodriguez-Moran et al. reported high HBA1C percentages and triglyceride levels in patients with diabetic foot ulcers, and low serum magnesium levels when compared to the controls; however, these results did not reach statistical significance.^17^^,^^26^

Our results suggest that low magnesium levels are not associated with the duration of diabetes and foot ulcers; although it is well known that complications of diabetes are strongly related to serum glucose levels and HBA1C percentages when compared to the duration of DM. Similarly, the Haquea et al. study concluded that the serum magnesium level has no direct relationship with the duration of DM.^27^

Consequently, we determined that low magnesium levels are associated with high glucose levels, high HBA1C percentages and low HDL levels. Uncontrolled diabetes and low HDL levels are important risk factors for atherosclerosis, which can lead to foot ulcers. Neuropathy, which is also a risk factor for foot ulcers, can develop from high glucose levels due to a magnesium deficiency.

Our study results are reinforced by previous studies, including Rodriguez-Moran et al., who reported that serum magnesium deficiencies were present in patients with type 2 DM, showing a strong relationship with foot ulcers.^26^ In that study, they compared 33 diabetic foot ulcer patients with 66 patients with DM without foot ulcers. In the Dasgrupta et al. study, a high incidence of foot ulcers in patients with hypomagnesemia was seen when compared to the controls (58.8% vs. 22.5%).^8^

To our knowledge, this is the first study that investigates the magnesium levels in three different groups (one study and two control groups). We investigated the magnesium levels in patients with diabetes and foot ulcers, patients with diabetes without foot ulcers and healthy subjects. Our study group is larger than previous studies, and we included more exclusionary criteria.

The present study had some limitations. First, the nutritional levels of all of the subjects were not the same; therefore, we could not know how much magnesium each participant received daily. Secondly, it would have been beneficial if the sample size had been larger. 

In conclusion, the magnesium levels should be controlled in patients with DM, with or without foot ulcers, and magnesium supplementation can be a complimentary treatment for these patients. In addition, magnesium supplementation can decrease the mortality rate in critically ill patients with type 2 diabetes mellitus.^28^

## Notes

***Source of founding:*** None.

## Notes

***Note:*** Potential conflicts do not exist.

Institution Review Board approval date/no: 11.07.2011/60
